# 

**Published:** 1889

**Authors:** 


					﻿REPERTORY
OF THE
CHARACTERISTIC SYMPTOMS,
CLINICAL AND PATHOGENETIC,
OF THE
Homoeopathic B|atei,ia IVJedica
EDITED BY
EDMUND J. LEE, M. D.
Published as a Supplement to The Homceopathic Physician,
Office, 1123 Spruce Street, Philadelphia, Pa.
||otice to our Subscribers.
The delay in publishing the combined issue of this
journal, for October and November, has been caused
by my inability to supervise and correct the Repertory
as it went through the press. The work was nearly
printed by the first of November, and would have
appeared had I not been compelled to cease all work
for some weeks.
The December issue has been held back, hoping
for the completion of the Repertory number. The
Repertory part is not yet ready, but will be mailed
to each subscriber as soon as I can complete it.
EDMUND J. LEE.
January 2p, 1890.
NOTICE.
All articles for publication, books for review, business communications, etc.,
must be sent to Editors, 1125 Spruce Street, Philadelphia.
Subscribers will please notice that this Journal will be sent until arrears
are paid and it is ordered discontinued.
				

